# Feline Leishmaniosis: An Emerging Public Health Problem

**DOI:** 10.3390/vetsci8090173

**Published:** 2021-08-30

**Authors:** Ana Elena Ahuir-Baraja, María Pilar Ruiz, María Magdalena Garijo, Lola Llobat

**Affiliations:** Department of Animal Production and Health, Veterinary Public Health and Food Science and Technology (PASAPTA), Facultad de Veterinaria, Universidad Cardenal Herrera CEU-CEU Universities, 46115 Valencia, Spain; ana.ahuir@uchceu.es (A.E.A.-B.); ruirodmar1@alumnos.uchceu.es (M.P.R.)

**Keywords:** cats, feline leishmaniosis, *Leishmania infantum*, zoonoses

## Abstract

Leishmaniosis is the third most important vector-borne disease in humans, preceded by malaria and lymphatic filariasis, and it is considered endemic in tropical and subtropical areas, where higher temperatures favor development of its vector, sandflies. This zoonotic disease is caused by infection of protozoa *Leishmania* spp. and the most serious mucocutaneous and visceral form is produced by *Leishmania infantum*, which predominates in the Mediterranean region. The usual hosts for this parasite are dogs and humans, but an increment in cases of *L. infantum* infection has been observed in cats in the last years. This increase could be due to the use of sandflies repellents in dogs, obligating the parasite to looking for other hosts. The role of cats in the epidemiology of this disease is unknown, although increase of prevalence of feline leishmaniosis has been observed in endemic areas in the last years. Diagnostic techniques and treatments in cats are not standardized, which makes it difficult to establish prevalence and epidemiology of feline leishmaniosis. Furthermore, the clinical signs and immune response against *Leishmania* in cats are different to those in dogs, with an observed increment of drug resistance. It is necessary to increase our knowledge about *L. infantum* infection in cats, including clinical signs, transmission, treatments, and the role of cats in the increasing of zoonoses. Finally, new alternative treatments are required for controlling the spread of this disease in all species of mammals.

## 1. Introduction

Leishmaniosis is a disease caused by the infection of protozoan parasite *Leishmania* spp. and transmitted by sandflies of the family Psychodidae (genus *Phlebotomus* in the Mediterranean region) [1,2,3,4]. The World Health Organization (WHO) estimates between 700,000 and 1,000,000 new cases in humans annually. This parasitosis is the third most important vector-born disease in humans, only preceded by malaria and lymphatic filariases, and it is considered endemic in tropical and subtropical areas, where the higher temperatures favor the development of sandflies [5]. Humans, together with domestic dogs (*Canis lupus familiaris*), are the main hosts, in which the diseases, caused by *Leishmania infantum*, represent an important problem for public health [6]. Infection in other animals, such as cats (*Felis catus*), wild canids, and horses, has been reported [7,8,9]. Although dogs used to be considered uniquely and mainly responsible for the spread of the disease to human, the increase in the number of cases diagnosed in domestic cats [10,11], and *L. infantum* parasites detected in cats sharing the same genetic characteristics with *L. infantum* strains isolated from humans and dogs [12,13,14], indicate that this species may play an important role currently in the epidemiology of infection in humans and dogs.

The first case of feline leishmaniosis (FL) was detected by Sergent et al. (1912) in Argelia [15]. FL cases have been described later in Europe, Latin America, and Asia, and their prevalence has increased considerably in recent years, with results of prevalence from 1.3% in Portugal or Qatar, to 22.5% and 25% in Brazil and Iran, respectively [10,16,17,18,19,20,21]. Although the highest prevalence of feline leishmaniosis has been found in countries where the disease is endemic, there are cases reported in other areas as well, such as the United States [22]. This FL rise could be connected with a host change due to the use of sandfly repellents in dogs, making them look for other hosts in which feed on [23,24]. In fact, the number of human leishmaniosis is also increasing, probably because the human companion animal bond is becoming higher with dogs and cats, rising the probability of infection [25]. Furthermore, some studies indicate that the use of secondary hosts by the parasite could be related to an increase in the virulence of *L. infantum* in humans. Concretely, human leishmaniosis outbreak in Spain with high virulence seems to be related to wild hares and wild rabbit’s infection. Both species were found to be asymptomatic reservoirs for the parasite in an area with a low dog population density [26,27]. Early detection of infection in dogs and cats, together with its surveillance and treatments, are strategies to control and avoid human infection, following the “One Health” concept. In addition, and considering cats as emergent hosts with a possible role in the spread of the disease, a new evaluation for the epidemiology and control in this species is necessary [6,28]. However, detection in cats is often confused with other infections, as the clinical signs of leishmaniasis in cats are nonspecific. Furthermore, in some cases, disease appears without clinical signs, making its detection and control more difficult. Moreover, immunosuppression provoked by viruses such as those causing leukemia or feline immunodeficiency can increase parasite multiplication [7]. Due to the scarce information about the role of cats in the distribution of the disease or as reservoir, it is necessary to carry out studies focused on FL, as it could also constitute a point of infection for humans.

In this review, the current knowledge about leishmaniosis in cats is summarized and revised. Comments about the detection and control techniques are included, as well as possible treatments against feline *Leishmania* spp. infections.

## 2. Epidemiology of Feline Leishmaniosis

Leishmaniosis is a zoonotic disease produced by parasites of genus *Leishmania*, mainly by the species *L. infantum* or *L. chagasi* in America [25]. Its principal host is domestic dogs (*Canis lupus familiaris*), but parasites have been isolated in rodents, lagomorphs, and wild canids, although the role of these species in the spread of the disease is not clear [8,26,29,30,31,32,33,34,35,36,37].

In cats, different species of *Leishmania* spp. have been identified, such as *L. infantum*, *L. mexicana*, *L. venezuelensis*, *L. braziliensis,* and *L. amazonensis* [25,38]. Recently, the first case of FL caused by *L. amazonensis* has been reported [39]. The transmission between host species is carried out through the bite of two genera of mosquitoes, *Phlebotomus* spp. and *Lutzomyia* spp. (*Psychodidae*) [19,25], but *L. infantum* has been isolated in fleas, ticks, and other arthropods, so they could also play an important role in the transmission even in cats [40,41]. Despite the fact that vertical and horizontal transmission are not well studied in felines [42], Vioti et al. (2021) have demonstrated by in vivo studies that infected cats are capable of transmitting *L. infantum* to sandflies [43]. Positive tests of *L. infantum* in cats have been reported in different countries in Europe and with different methods in the last twenty years (Table 1), showing the increasing relevance of cats in the transmission of the disease. Moreover, the existence of asymptomatic infection by *L. infantum* in apparently healthy stray cats has been demonstrated in Spain [44], which increases the importance of carrying out studies in cats as transmitters of the infection, mainly in endemic areas.

Different prevalence of infection according to sex in non-neutering animals has never been observed, but factors such as age; neutering status; or co-infection with viruses as feline immunodeficiency virus (FIV) or feline leukemia virus (FeLV), mycoplasmas or other parasites, including *Toxoplasma gondii* (Protozoa), seem to be considered a determinant factor [53,56,57,58]. Table 2 summarizes the different organisms, including bacteria, viruses, and other protozoan detected in co-infection with *L. infantum* in cats.

## 3. Immune Response and Clinical Signs in Cats

**Figure 1:**
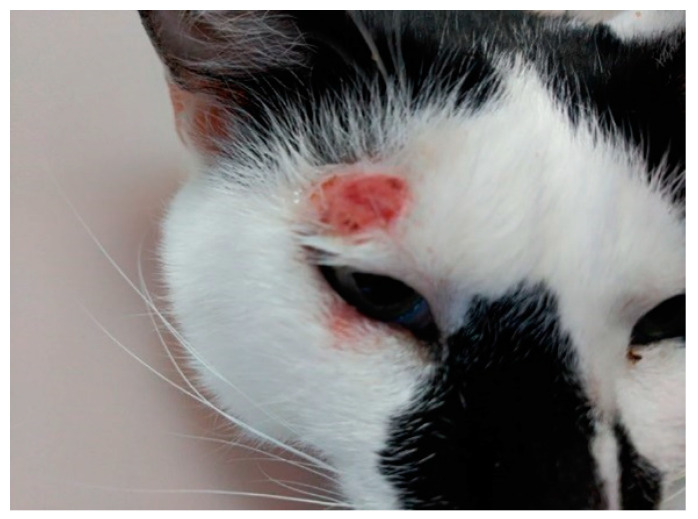
Ulcerative lesion in the temporal area of head related to *L. infantum* feline infection.

First system of defense in all mammals is physic-chemical barriers, which are not efficient against *Leishmania* spp. infections, allowing sandfly mouthparts to pass through the skin injecting promastigotes. After that, the immune system and, more specifically, innate immunity, such as macrophages, neutrophils, and natural killer (NK) cells, are the first mechanisms against infection [65,66,67]. The parasite has the ability to survive phagocytization by macrophages, where promastigotes develop into amastigotes [68]. Specific immunity consists in Th1 and Th2 lymphocytes as well as antibodies production, which are unable to eliminate the parasites and can even favor its growth and proliferation. Antibodies are deposited in kidneys, causing glomerulonephritis, and subsequent kidney failure [38]. Contrary to what happens in dogs, a high number of antibodies in cat blood is not related with positive PCR. This explains the high number of cats without clinical signs, which can show skin lesions before they produce antibodies, and low prevalence of kidney failure. Additionally, in most cases of FL, spontaneous remission of clinical signs is frequent, which may be due to the Th1 immune response that gives rise to seroconversion followed by clinical resolution [69]. While in dogs leishmaniosis is a chronic and progressive disease which affects all tissues and organs, in cats this pathology affects commonly spleen, liver, lymph nodes, bone marrow, kidney, and eyes, dermatological and mucocutaneous being the most frequent clinical forms (Figure 1) and more frequent than dogs [23,70,71,72,73]. In fact, a recent systematic review summarizes the skin lesions in feline leishmaniosis, indicating that the most reported clinical signs were nodules, followed by ulcers, which were less frequently reported [74]. The most frequent clinical and clinicopathological abnormalities reported in feline leishmaniosis were skin and/or muco-cutaneous nodules and ulcers, and lymphadenomegaly [74].

Actually, clinical signs in cats could be due to co-infection with immunosuppressive disease such as cancer or viruses infection [38,42]. Thus, feline immunodeficiency (FIV) or feline leukemia (FeLV) viruses may favor the development of FL. Correlation with leishmaniosis has not been demonstrated yet in cats, although a correlation with similar pathologies as HIV in humans and dogs is well documented [70,75,76]. Different prevalence of *L. infantum* infection in dogs and cats agrees with the fact that cats seem to present low reaction to parasites [61]. Co-infections with *Hepatozoon felis* (protists) and *Candidatus mycoplasma* haemominutum (bacteria) have been also reported [59] (Table 1). Other diseases that can be related with the improvement in the detection of clinical signs of FL are carcinomas, for which correlation in humans and dogs has been demonstrated [77]. Maia et al. (2015) presented for the first time a clinical report of FL associated with an invasive squamous cell carcinoma in a cat [24].

On another hand, different prevalence of *L. infantum* infection in dogs and cats could be explained because cats seem to present low reaction to parasites. Recently, higher blood parasite burdens have been observed in dogs than in cats with the same exposition to sandflies. Nevertheless, seroactivity in cats was higher than in dogs, so it seems cats are less effective in transmitting the disease [55].

## 4. Diagnosis of FL

Laboratory analyses to direct or indirect diagnosis of leishmaniosis are made by serological, parasitological, and molecular techniques [24,78]. Serological tests may be not sensitive enough, so it is necessary to combine them with other techniques such as parasitological or molecular methods in cats [24]. Iatta et al. (2020) have recently developed a new immunofluorescence antibody test with good results for the detection of *L. infantum* infection in cats [79]. In clinical practice, satisfactory results have been obtained for FL detection by polymerase chain reaction (PCR), as it occurs in the detection of other diseases like feline herpes virus [28]. Molecular techniques, as PCR, have high sensibility to confirm presence of *L. infantum*, but detection of DNA does not implicate infection in cats [38]. Direct immunofluorescent assay (DIA) has been applied for diagnosing visceral leishmaniosis in cats, using the same cut-off as for dogs, but the results obtained are probably underestimating the infections in cats, as the cats present lower antibody values than dogs [80]. Enzyme-linked immunosorbent assay (ELISA) is also used for diagnosis of FL, showing discrepant results compared with other techniques, which suggest that it should be combined with other parasitological and/or molecular techniques [81]. The immunological diagnostic methods recommended by LeishVet Guideline (2018) were IFAT with cut-off of 1:80, ELISA, DAT, with cut-off of 1/800, Western blot where a 18 kDa band should be detected [74]. As for dogs, interferon gamma (IFN-γ) analysis in cat’s blood could provide a better estimation of cat exposure to the disease when it is associated to serological and molecular tests [48,82]. Recently, Savioli et al. (2021) and Urbani et al. (2020) showed that elevated levels of amyloid A, gamma globulins, and alpha 2 globulins could be a good indicator of disease in cats [83,84], although more studies will be necessary to find definitive markers of disease.

Most epidemiological studies have been carried out on visceral leishmaniosis diagnosis and only some of them have analyzed cutaneous or mucocutaneous leishmaniosis. In the latter form, ELISA and indirect fluorescent antibody (IFA) methods are the most sensible for the diagnosis, along with Western blotting (WB), as they present between 97% to 100% of specificity [78]. Persichetti et al. (2017) compared ELISA, IFA, and WB techniques and concluded that ELISA is more adequate for the diagnosis of clinical leishmaniosis, but IFA and WB are more sensible methods and, therefore, they should be used for the diagnosis of the subclinical forms [81].

When cutaneous lesions are present (Figure 1), fine-needle aspiration puncture from the skin nodules can be helpful to determine granulomatous inflammation, typically produced by *Leishmania* spp., and observed in ocular lesions of cats. Ear and nasal planum lesions are considered as differential diagnoses of squamous cell carcinoma [69,70,71,85]. Without cutaneous lesions, cats could present granulomatous rhinitis [86]. Histiocytic infiltration of tissue, a typical sign of canine leishmaniosis, is not a specific marker in FL [87]. In visceral leishmaniosis, cytology is useful to detect lymphoid hypoplasia in spleen, with the presence of amastigotes, inflammatory infiltrated liver mainly composed of macrophages in the portal area and between hepatocytes [69,88]. In summary, clinical signs of FL are similar to those shown in canine leishmaniosis, but with specific features, such as ulcerative and nodular skin lesions [72,89]. Additionally, a case of FL with inflammatory mammary gland fluid has been described [90]. Since many of the clinical signs showed in infected cats are due to comorbidities (Table 1), it is necessary to try to differentiate clinic-pathological abnormalities due to FL. In fact, these co-infections can be present in around 10% of infected cats by *L. infantum* [91]. Chatzis et al. (2020) have recently published a study to determine clinical signs of FL, assessing whether the disease is associated with laboratory abnormalities analysis [47]. Diagnosis of FL is clearly difficult, and all these data could explain the high number of infected asymptomatic cats, increasing its importance as a vector for transmitting the parasite to humans.

The identification of *Leishmania* spp. is also very important for diagnosis, treatment, and management of the disease. However, it is not always easy, since molecular techniques are needed, based on high resolution melting or specific markers analysis, to improve results [11,92].

## 5. Possible Treatments and Control of Disease

Approximately half of the cats infected by *Leishmania* spp. may recover spontaneously, although the studies about the specific treatment are limited, and the actual efficacy of the treatments is unknow [7]. Administration of allopurinol seems to improve clinical signs, but it is not yet known if it may cause relapses as it does in dogs [22,93,94]. The LeishVet Guidelines (2018) recommended treatment of allopurinol 10 mg/kg 12 h, or 20 mg/kg 24 h, for at least 6 months [74]. Other treatments combine allopurinol with meglumine antimoniate, but some authors indicate that only the latter presents a good clinical response in cats [38,42]. Nonetheless, lower effects of antimonials, as meglumine antimoniate, have been observed due to the increase of *Leishmania* spp. drug resistance, even though the mechanism of resistance remains unclear [95]. Flavonoids as fisetin have also been tested and it seems that they prevent promastigotes growth in culture [96].

Considering there is currently no definitive treatment, either for dogs or cats, prevention is essential to reduce the prevalence of this zoonosis, especially in endemic areas. The use of repellents is the most common method for preventing leishmaniosis in dogs. Nevertheless, usual repellents as pyrethrins and pyrethroids are toxic to cats [97]. Nowadays, the unique method for prevention in cats is the use of a polymer collar composed by 10% imidacloprid and 4.5% flumethrin. It shows a 75% of efficacy against FL, and it also protects against ticks, fleas, and other arthropods [98]. In regard to vaccines, while there are one or two (depending of country) commercial ones against leishmaniosis in dogs, none have been synthetized for FL [99,100]. Factors such as cleaning of environment where cats live and avoiding outdoor access could be helpful in the prevention of *L. infantum* infection in cats [52].

## 6. Conclusions

The use of sandfly repellents in domestic dogs (*Canis lupus familiaris*) for avoiding leishmaniosis has caused the parasite to hunt for other mammalian hosts. Given that there is evidence of an increase in the virulence of the parasite when infecting a secondary host, it is of great relevance for the global health of both animals and humans to control and eradicate *Leishmania* spp. infection in all species of mammals. One of these new target hosts are domestic cats (*Felis catus*), in which an increase of leishmaniosis by *L. infantum* infection has been observed in recent years, mainly in areas where the disease is endemic. Epidemiology and prevalence of infection in cats is little known and the results are very different, depending on the technique used for the diagnosis or the geographical area. Regardless of this disparity in the data collected so far on the prevalence of infection in cats, an increase in cases is beginning to be worrying, especially since cats are the second most frequent pet in the world. On the other hand, there is little knowledge about clinical signs of the disease in cats and these cannot be compared to those found in dogs, since the two species present significant differences in their immune response to pathogens, such as the antibody titers in blood. In this way, the usual clinical practice applied in dogs is not useful for infected cats. In addition, around half of infected cats recover spontaneously, so the danger of this species as a reservoir for the parasite increases. The fact that we cannot use the same repellents as in dogs, and the increase of drug resistant parasites, makes it necessary to carry out more studies to expand the knowledge on clinical and preventive aspects to reduce or eradicate this zoonosis in cats.

## Figures and Tables

**Table 1:** Countries where high positive percentage of cases of *L. infantum* infection in cats have been reported and detection method (IFAT: immunofluorescence antibody test; DAT: direct agglutination test; PCR: polymerase chain reaction; ELISA: enzyme-linked immunosorbent assay). The material analyzed was serum samples for IFAT and ELISA tests, and whole blood for PCR test. * In this study, the authors analyze the antibodies to *Leishmania* spp.

Country	Method of Detection	Positive % Founded	References
Albania	IFAT and PCR	0.7	[45]
Cyprus	ELISA and PCR	5.8	[46]
Greece (Macedonia and Thessaly)	IFAT, ELISA and PCR	46.0	[47,48]
Italy (Sicily)	ELISA and PCR	36.0	[49]
Portugal (Lisbon)	IFAT and PCR	20.4	[24]
Portugal (Madeira Island)	DAT	0.0	[50]
Spain (South)	IFAT and PCR	48.3	[7]
Germany	IFAT and PCR	4.0	[51]
Qatar (Doha)	PCR	1.3	[21]
Brazil (Amazon region)	IFAT	30.5	[52]
Angola (Luanda) *	DAT	3.9	[53]
Iran (Kerman)	PCR	13.9	[54]
Israel	ELISA	75.0	[55]

**Table 2:** Organisms (including bacteria, viruses, and protists) founded in co-infection with *L. infantum* in cats.

Organisms	Reference
*Hepatozoon felis* and *Candidatus mycoplasma* haemominutum	[59]
*Toxoplasma gondii*	[53,60]
Feline immunodeficiency virus (FIV) and feline leukemia virus (FeLV)	[56,61]
*Mycoplasma* spp., FIV and FeLV	[57]
*Toxoplasma gondii* and FIV	[58]
*Rickettsia felis*	[62]
*Ehrlichia* spp. and *Bartonella* spp.	[63]
*Babesia* spp. (only in wild cats)	[64]
*Hepatozoon* spp. (only in wild cats)	[64]

## Data Availability

Not applicable.
